# Recent Advances in Engineering Nanomedicines for Second Near-Infrared Photothermal-Combinational Immunotherapy

**DOI:** 10.3390/nano12101656

**Published:** 2022-05-12

**Authors:** Fengshuo Wang, Jingyi Zhu, Yongtao Wang, Jingchao Li

**Affiliations:** 1 Shanghai Engineering Research Center of Nano-Biomaterials and Regenerative Medicine, College of Chemistry, Chemical Engineering and Biotechnology, Donghua University, Shanghai 201620, China; 18438871231@163.com; 2 School of Pharmaceutical Sciences, Nanjing Tech University, Nanjing 211816, China; zhujy1210@njtech.edu.cn; 3 School of Medicine, Shanghai University, Shanghai 200444, China

**Keywords:** second near-infrared windows, immunotherapy, optical nanomedicines, photothermal therapy, combinational therapy

## Abstract

Immunotherapy has emerged as one of the major strategies for cancer treatment. Unlike conventional therapeutic methods, immunotherapy can treat both primary and distant metastatic tumors through triggering systematic antitumor immune responses and can even prevent tumor recurrence after causing the formation of immune memory. However, immunotherapy still has the issues of low patient response rates and severe immune-related adverse events in clinical practices. In this regard, the combination of nanomedicine-mediated therapy with immunotherapy can modulate a tumor immunosuppressive microenvironment and thus amplify antitumor immunity. In particular, second near-infrared (NIR-II) photothermal therapy (PTT), which utilizes light conversions to generate heat for killing cancer cells, has shown unique advantages in combining with immunotherapy. In this review, the recent progress of engineering nanomedicines for NIR-II PTT combinational immunotherapy is summarized. The role of nanomedicine-mediated NIR-II PTT in inducing immunogenic cell death and reprogramming the tumor immunosuppressive microenvironment for facilitating immunotherapy are highlighted. The development of NIR-II-absorbing organic and inorganic nonmetal and inorganic metal nanomedicines for the NIR-II PTT combinational immunotherapy of cancer is also introduced in detail. Lastly, the current challenges and future perspectives of these nanomedicines for combinational immunotherapy are proposed.

## 1. Introduction

In recent years, immunotherapy has become another effective treatment strategy for cancer after surgery, radiotherapy, chemotherapy, and targeted therapy [1,2,3]. Immunotherapy that trains the host antitumor immune responses can eliminate both local tumors and distant metastases, as well as trigger long-term immune memory to prevent tumor recurrence [4,5,6]. Currently, cancer vaccines, chimeric antigen receptor T-cell therapy, and immune checkpoint blockade therapy are the three main strategies for immunotherapy [7,8,9]. A large number of immunotherapeutic drugs have been approved for the treatment of different malignant tumors, leading to effective tumor treatment in a subset of patients [10,11,12,13]. However, there are two major challenges for clinical applications of immunotherapy [14,15,16]. First, the response rates of patients for immunotherapy are low, which results in limited therapeutic efficacy [17,18,19]. For example, only around 10–30% tumors in clinical patients can be effectively treated by immune checkpoint blockers, while most of the tumors respond poorly to immune checkpoint blockade therapy because of their low immunogenicity [20,21,22,23]. Second, immunotherapy has the possibility to cause immune-related adverse events, such as diabetes mellitus, myocarditis, thyroid dysfunction, hypophysitis, and hypokalemia, particularly for high-dosage injections and/or combinations of multiple immunotherapeutic drugs [24,25,26,27,28]. Therefore, it is highly desired to develop effective and safe approaches to achieve ideal antitumor immune responses.

Photothermal therapy (PTT) utilizes the light irradiation of photothermal agents to generate heat for cancer cell killing. PTT has shown a great promise for cancer treatment because of the noninvasiveness, high spatiotemporal precision, simple operation, and flexible tenability of light sources [29,30,31,32]. Thus, PTT often shows high selectivity and specificity for cancer treatment without causing obvious damage to normal tissues [33,34,35,36]. The main light sources used for PTT are the first NIR light (NIR-I, 650–950 nm), which however, has limited tissue penetration depth (less than 1 cm) and relatively low maximum permissible exposure for skin [37,38,39]. Therefore, the extensive applications of PTT have been greatly hindered. To address these issues, a new optical window termed the second NIR (NIR-II) window (1000–1700 nm), with better tissue penetration depth (around 3–5 cm) and higher maximum permissible exposure relative to NIR-II light, has been adopted for NIR-II PTT [40,41,42]. Such advantages of NIR-II light not only enable the treatment of deep regions of tumors but also allow for strong power density to improve heat generation, thus achieving high PTT therapeutic efficacy [43]. To date, different nanosystems such as metallic nanoparticles, metal–organic hybrid nanoparticles, inorganic semiconducting nanoparticles, organic polymer nanoparticles, and small-molecule-based nanoparticles have been developed for NIR-II PTT [44,45,46,47,48].

In addition to the direct ablation of tumors, NIR-II PTT has been recently used to reprogram the tumor immunosuppressive microenvironment to potentiate cancer immunotherapy [49]. The generated heat during NIR-II PTT can induce immunogenic cell death (ICD) of cancer cells, which is characterized by the release of tumor-associated antigens, adenosine triphosphate, and high mobility group box 1 protein into the extracellular environment, and the translocation of calreticulin to the cell surface [19]. Such an action can promote the uptake and processing of antigens by antigen presentation cells and facilitate the production and priming of effector T cells, leading to the activation of antitumor immunity for the eradication of tumors and metastases [50]. In addition, the NIR-II PTT-mediated ICD effect can further enhance antitumor immune responses of immunotherapy, leading to combinational action for effective treatments of tumors [51].

In this review, the recent progress of engineering nanomedicines for NIR-II PTT combinational immunotherapy is summarized. The development of NIR-II-absorbing organic and inorganic nonmetal and inorganic metal-based nanomedicines, and their uses for inducing ICD and reprogramming the tumor immunosuppressive microenvironment to mediate NIR-II PTT combinational immunotherapy with greatly improved efficacy, are introduced in the following sections. Finally, a brief summary, existing challenges, and future perspectives in this field are discussed.

## 2. Organic Nanomedicines for NIR-II PTT Combinational Immunotherapy

### 2.1. Small-Molecule-Based Nanoparticles

Some organic small-molecule-based nanoparticles with strong NIR-II absorbance and excellent photothermal conversion efficacy have been constructed for NIR-II PTT combinational immunotherapy. In a recent study of our group, 3,3′,5,5′-tetramethylbenzidine (TMB)-based liposome nanocomplexes with pH-responsive NIR-II photothermal properties were constructed for combinational immunotherapy [52]. Thermal-responsive liposomes containing an amphiphilic polymer, 1,2-distearoyl-sn-glycero-3-phosphoethanolamine-poly(ethylene glycol) (DSPE-PEG), and thermal-responsive 1,2-dipalmitoyl-sn-glycero-3-phosphocholine (DPPC), with a transition temperature of 41 °C, were synthesized to load pH-sensitive charge-transfer nanoparticles as the NIR-II photothermal agent, deoxyribonuclease I (DNase I), and stimulants of the natural killer (NK) cell (SIS3). Charge-transfer nanoparticles were transformed from TMB and exhibited a stronger absorption in the NIR-II window in an acidic environment relative to neutral and basic conditions and thus have pH-sensitive NIR-II photothermal properties [53]. Such nanocomplexes exerted a NIR-II PTT effect under 1064 nm laser irradiation (1.0 W/cm^2^, 6 min) in a tumor acidic microenvironment, resulting in the increase of tumor temperature at around 45 °C. This destroyed the thermal-responsive liposomes to allow the on-demand release of SIS3 and DNase I. Due to the toxicity of DNase I, cancer cells were killed, and ICD was induced to promote immune responses. The action of SIS3 could synergize with DNase I-mediated ICD to promote the activation of NK cells and CD8^+^ cytotoxic T lymphocytes (CTLs). Thus, such a combinational immunotherapy could effectively inhibit the growth of primary and distant 4T1 tumors and completely prevented lung metastasis in subcutaneous mouse models.

**Figure 1:**
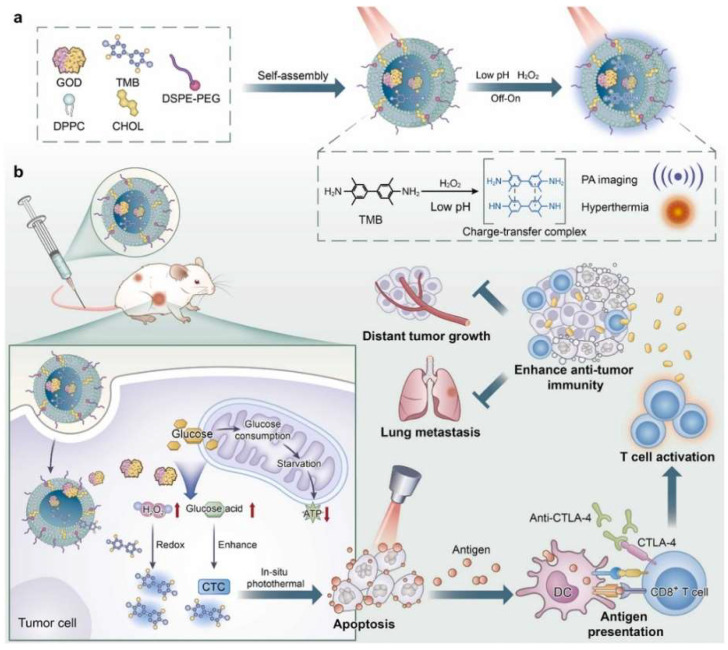
(**a**) A schematic illustration of the fabrication of a liposome-based cascade nanoreactor (LGT) containing TMB and GOD. (**b**) A schematic illustration of the NIR-II laser-triggered in situ NIR-II PTT and the starvation combinational immunotherapy. Reproduced with permission from [54]. Copyright 2022, Elsevier.

In another study, a liposome-based cascade nanoreactor containing TMB was reported for NIR-II PTT combinational starvation and immunotherapy [54]. The cascade nanoreactors (denoted as LGT) were constructed by encapsulating TMB as the NIR-II photothermal agent and glucose oxidase (GOD) serving as a tumor starvation mediator into liposomes composed of DPPC, DSPE-PEG, and cholesterol (CHOL) (Figure 1a). Due to the GOD-mediated depletion of glucose and the production of hydrogen peroxide (H_2_O_2_) in tumor cells, TMB was in situ converted into the NIR-II-absorbing charge transfer complex for NIR-II PTT and photoacoustic imaging. The depletion of glucose not only cut the supply of energy to the tumor cells for starvation therapy but also reduced the adenosine triphosphate levels to enhance the PTT effect of the LGT, leading to cell apoptosis and TAA release under 1064 nm laser irradiation (1.5 W/cm^2^, 10 min) (Figure 1b). Thus, a vaccine-like immune response was caused after the treatment of primary tumors via combinational NIR-II PTT and starvation therapy, which could be further combined with the anti-cytotoxic T lymphocyte antigen-4 (anti-CTLA-4) antibody-mediated immune checkpoint blockade therapy to suppress distant tumor growth and lung metastasis in 4T1 tumor-bearing-mouse models.

The combination of reactive oxygen species (ROS) and NIR-II PTT-mediated heat generation for ICD induction and the promotion of immunotherapy has also been reported. As shown in Zhao’s group, small-molecule-based organic metal adjuvants (OMAs) with NIR-II photothermal properties and ROS-generating ability were developed for cancer therapy [55]. Such OMAs were constructed through the supramolecular assembly of commercially available donors and acceptors, showing excellent NIR-II photothermal properties and photoacoustic imaging performance via optimizing the constituting components. In a tumor microenvironment, OMAs oxidized cysteine and glutathione (GSH) to inhibit the biosynthesis of GSH, resulting in the disruption of redox homeostasis and boosting ROS accumulation inside cells. Under 1064 nm laser irradiation (1.0 W/cm^2^, 5 min), OMAs exerted an NIR-II PTT effect to ablate cancer cells. In addition, the PTT effect and ROS generation mediated ICD induction with improved efficacy to enhance the immune response by promoting the maturation of dendritic cells (DCs) and the infiltration of T cells. Such an ROS-generating NIR-II PTT could be combined with anti-programmed cell death protein 1 (aPD-1) antibody-mediated immune checkpoint blockade therapy to allow for the increased infiltration of T cells into tumor tissues, leading to the eradication of primary tumors and the significant inhibition of distant tumor growth in 4T1 tumor-bearing-mouse models.

To boost ROS generation for combining NIR-II PTT and immunotherapy, Shen’s group reported an “all-in-one” hydrogel for cancer treatment [56]. Such hydrogels were formed by loading ink as the NIR-II photothermal agent, HY19991 as the PD-L1 inhibitor, and an azo-initiator of 2,2-azobis [2-(2-imidazoline-2-acyl)propane]dihydrochloride (AIPH) into alginate hydrogels in situ crosslinked with Ca^2+^. Under 1064 nm laser irradiation (0.5 W/cm^2^, 10 min), the ink exerted NIR-II PTT to increase the local temperature at around 45 °C, leading to the upregulation of PD-L1 expression and the formation of a large number of alkyl radicals from AIPH. The formed alkyl radicals augmented the ICD effect and increased the recruitment of tumor-infiltrating lymphocytes into tumors via the hydrogel-mediated conversion of “cold” tumors into “hot” tumors. Moreover, hydrogels released HY19991 to block the binding between PD-L1 and PD-1 to further improve the antitumor immunity. As a result, such “all-in-one” hydrogels could afford synergistic action via a mild PTT effect to reverse the tumor immunosuppressive microenvironment and triggered both innate and adaptive immune responses in CT26 tumor-bearing-mouse models, leading to the effective elimination of tumors and the prevention of distant metastatic tumors.

### 2.2. Semiconducting Polymer Nanoparticles (SPNs)

SPNs, as a class of optical materials transformed from semiconducting polymers (SPs) with excellent optical properties and biocompatibility, have also been used for NIR-II PTT and thus can mediate NIR-II PTT combinational immunotherapy [57,58,59]. To enhance the therapeutic efficacy of SPNs, Zhang and Pu’s groups developed a polymer multicellular nanoengager for synergistic NIR-II photothermal immunotherapy [60]. The nanoengager consisted of NIR-II-absorbing SPs as the photothermal agents and the surface camouflaged cell membranes derived from DCs and immunologically engineered tumor cells serving as the cancer vaccine shells. Such a design enabled multicellular engagements among T cells, DCs, and tumor cells, resulting in the enhanced activation of DCs and T cells. These nanoengagers could effectively accumulate into both lymph nodes and tumor tissues after systemic administration and acted as nanovaccines to trigger the immune response. Under 1064 nm laser irradiation (1.0 W/cm^2^, 10 min), the nanoengagers exerted NIR-II PTT to eradicate tumors and induce ICD for further eliciting antitumor T cell immunity. The nanoengager-mediated NIR-II PTT synergistic immunotherapy not only efficiently inhibited the growth of both primary and distant 4T1 tumors and eliminated tumor recurrence but also triggered immunological memory for long-term immune surveillance.

**Figure 2:**
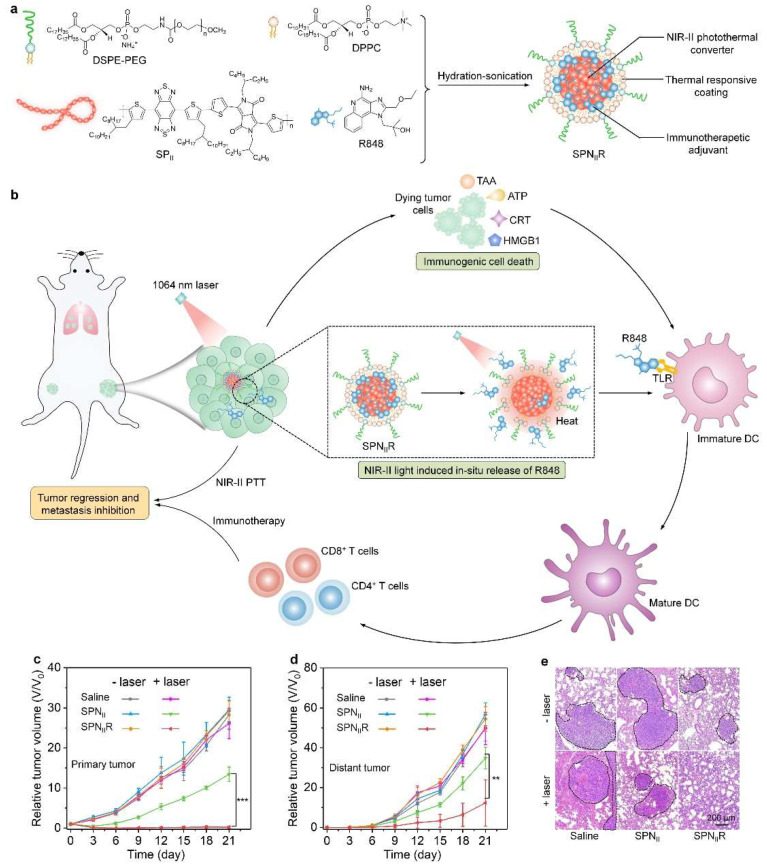
(**a**) The chemical structures of DSPE-PEG, DPPC, SP_II_, and R848, and the synthesis of SPN_II_R. (**b**) A schematic illustration of the mechanism of SPN_II_R for NIR-II synergetic photothermal immunotherapy. (**c**) The relative tumor volumes of the primary tumors from 4T1 tumor-bearing BALB/c mice after the systemic administration of saline, SPN_II_, or SPN_II_R through tail-vein injection with or without laser irradiation at 1064 nm. (**d**) The relative tumor volumes of distant tumors from 4T1 tumor-bearing BALB/c mice after different treatments. (**e**) Hematoxylin and eosin (H&E) staining images of lung metastasis from mice after different treatments for 21 days. Reproduced with permission from [61]. Copyright 2021, Wiley-VCH. Data are expressed as mean ± SD, ** *p* < 0.01, *** *p* < 0.001.

The SPN-mediated NIR-II photothermal effect has also been used to achieve the on-demand release of immunotherapeutic drugs in target tumor tissues for safe and effective immunotherapy. For example, we recently constructed SP-based nanoadjuvant-possessing NIR-II photothermal properties for cancer immunotherapy [61]. An SPN serving as the NIR-II photothermal agent and a toll-like receptor (TLR) agonist acting as the immunotherapeutic adjuvant were loaded in the thermal-responsive liposomes to form nanoadjuvants (termed as SPN_II_R) (Figure 2a). Under 1064 nm laser irradiation (1.0 W/cm^2^, 5 min), SPN_II_R exerted NIR-II PTT, leading to the direct ablation of tumor cells, the induction of ICD, and the in situ release of the TLR agonist (R848) through the breaking of the structure of the thermal-responsive liposomes (Figure 2b). The ICD action and the released R848-mediated TLR activation could promote the maturation of DCs, thereby enhancing the activation of T cells. As such, the antitumor immunity was obviously improved as the populations of CD4^+^ and CD8^+^ T cells and the secretions of immune-relevant cytokines in tumors were increased after SPN_II_R-mediated treatment. The SPN_II_R-mediated NIR-II PTT and immunotherapy displayed high efficacy in absolutely eradicating primary tumors, effectively inhibiting distant tumors, and suppressing lung metastasis in 4T1 tumor-bearing-mouse models (Figure 2c–e).

By using thermo-responsive linkers, Pu’s groups reported an activatable polymer nanoagonist for the NIR-II photothermal immunotherapy of cancer [62]. The nanoagonists were constructed by covalently conjugating R848 onto NIR-II-absorbing SPNs via a labile thermo-responsive linker. Under 1064 nm laser irradiation (1.0 W/cm^2^, 10 min), the nanoagonists exerted NIR-II PTT for killing tumor cells and inducing ICD. The generated heat also destroyed thermo-responsive linkers to achieve the on-demand release of R848 even in deep solid tumor tissue. The antitumor immune response in 4T1 tumor-bearing-mouse models was potentiated due to the combinational action of the NIR-II PTT-mediated ICD effect and R848-mediated TLR activation. Therefore, through the combinational action of NIR-II PTT and immunotherapy, these nanoagonists not only almost completely eradicated the primary tumors after direct laser irradiation but also obviously inhibited the growth of distant tumors and suppressed lung and liver metastasis.

In another study, Pu and coworkers reported a NIR-II light-activatable polymeric nanoantagonist for photothermal immunometabolic cancer therapy [63]. The polymeric nanoantagonist was obtained by conjugating an adenosine A2A receptor antagonist (vipadenant) onto NIR-II-absorbing SPs via the thermo-responsive linkers. Under 1064 nm laser irradiation (1.0 W/cm^2^, 10 min), SPNs within nanoantagonists mediated NIR-II PTT to induce tumor thermal ablation and subsequently ICD, and the release of vipadenant through triggering the cleavage of the thermo-responsive linkers. The released vipadenant could block the binding between extracellular adenosine with A2A receptors on the surface of the CTLs and the regulatory T (T_reg_) cells, thus promoting the priming and infiltration of CTLs but suppressing the functions of the T_reg_ cells to achieve an enhanced antitumor immune response. Such a combinational action of NIR-II PTT and immunotherapy, which was mediated by these nanoantagonists, allowed for the complete eradication of primary 4T1 tumors, the effective inhibition of metastasis, and the prevention of tumor relapse after reinoculation.

## 3. Inorganic Non-Metal Nanomedicines for NIR-II PTT Combinational Immunotherapy

Some inorganic non-metal nanomaterials possessing an intrinsic nature of strong absorbance in the NIR-II window and good photothermal conversion efficacy can produce heat under NIR-II laser irradiation and thus are extensively expanded to manage the synergistic tumor treatment of PTT and immunotherapy. Xing’s group developed an immunoadjuvant-modified nanotube platform to accomplish the NIR-II PTT combinational immunotherapy of mouse tumors [64]. Single-walled carbon nanotubes (SWNTs) prepared by exfoliating pristine SWNTs under an ultrasonic process were mixed with glycated chitosan (GC) to construct the immunological SWNT-GC nanoplatforms. SWNT-GC not only maintained the optical and photothermal properties of SWNTs and the immunological functions of GC but also could be easily internalized into cells for efficient PTT treatment and triggering an immune response under NIR-II laser irradiation (0.75 W/cm^2^, 10 min). For tumor immunogenicity, GC acted as damage-associated molecular pattern molecules (DAMPs) and pathogen-associated molecular pattern molecules (PAMPs) for the enhanced presentation of antigens to reinforce the antitumor immunity. SWNT-GC treatment with laser irradiation afforded remarkably improved efficacy in suppressing tumor growth, increasing long-term mouse survival, and inhibiting tumor rechallenge using EMT6 tumor-bearing-mouse models.

**Figure 3:**
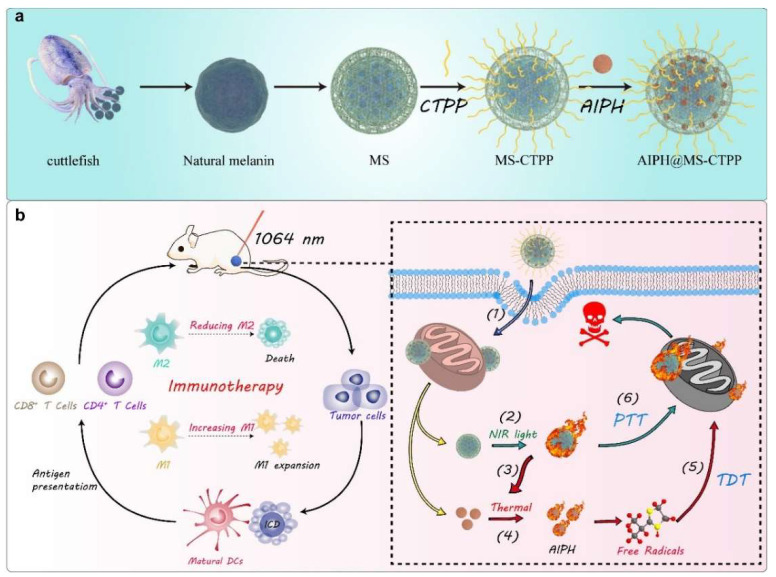
(**a**) Schematic illustrations of the construction of the AIPH@MS-CTPP nanostructures. (**b**) Schematic illustrations of the inhibition of tumor growth by AIPH@MS-CTPP-mediated synergizing NIR-II PTT, thermodynamic therapy, and immunotherapy. Reproduced with permission from [65]. Copyright 2022, Elsevier.

To obtain ideal antitumor efficacy, it is necessary to develop nanomedicines with targeting ability and multiple therapies. Recently, Wang’s group reported mitochondrial targeted melanin@mesoporous silicon dioxide (mSiO_2_) yolk-shell nanostructures for NIR-II-driven PTT, thermodynamic therapy, and immunotherapy [65]. Melanin nanoparticles from cuttlefish ink were coated with SiO_2_ to pack azodiisobutylimidazoline hydrochloride (AIPH@MS) and further surface-modified with (3-carboxypropyl) triphenylphosphonium bromide (CTPP) to form the multifunctional AIPH@MS-CTPP nanostructures (Figure 3a). These AIPH@MS-CTPP nanostructures showed enhanced accumulation and retention in the tumor tissues and were further delivered to thermally susceptible mitochondria due to the surface modification of CTPP. Under 1064 nm laser irradiation (1.0 W/cm^2^, 5 min), melanin nanoparticles within nanostructures mediated NIR-II PTT to produce heat, which not only resulted in direct death of tumor cells but also triggered the release of AIPH to generate oxygen-independent alkyl free radicals for thermodynamic therapy and further killing of tumor cells (Figure 3b). As a result, the melanin nanoparticle-mediated NIR-II PTT and AIPH-enabled thermodynamic therapy had a collaborative effect and induced local antitumor immunity; they effectively reprogrammed the M2 tumor-associated macrophages into the M1 phenotype. Such a therapy could be further combined with the anti-PD-1-mediated immune checkpoint blockade therapy, leading to increased populations of the CD4^+^ and CD8^+^ T cells and the M1 macrophages but reduced populations of the T_reg_ cells and the M2 macrophages in tumor tissues. Therefore, the growth of tumors and metastasis in 4T1 tumor-bearing mice was obviously inhibited.

## 4. Inorganic Metal Nanomedicines for NIR-II PTT Combinational Immunotherapy

Inorganic metal nanoparticles can be employed to gain efficient NIR-II PTT efficacies due to low immunogenicity and high thermal conversion efficacy [66,67,68]. Thus, inorganic metal nanomedicine-mediated NIR-II PTT is widely used for tumor ablation, targeted cancer cell death, and stimulating the immune response via triggering ICD. In recent years, different inorganic metal nanomedicines have been designed and developed to allow for the combinational action of NIR-II PTT with immunotherapy.

### 4.1. Gold Nanoparticles

Because of low cytotoxicity, the easy manipulation of geometry, and thermal-sensitive hyperthermia, gold nanoparticles have been extensively applied in NIR-II-induced PTT and immunotherapy. Chen and coworkers reported bovine serum albumin bioinspired gold nanorods (AuNRs) loaded with an immunoadjuvant for the synergistic therapy of PTT and immunotherapy in the NIR-II biowindow [69]. Cetyltrimethylammonium-bromide-coated AuNRs were successfully customized by bovine serum albumin and PEG. In addition, the immunoadjuvant imiquimod (R837) was incorporated into the formed AuNRs for cancer treatment. The obtained bifunctional AuNRs not only increased the temperature to 60 °C under 1064 nm laser irradiation (1.0 W/cm^2^, 10 min) to precipitate PTT but also promoted DC maturation for the antigen presentation and priming of T cells to activate the immune response for inducing cancer cell death. After the treatment of NIR-II PTT and immunotherapy, B16F10 tumors were destroyed, lung metastasis was inhibited, and long-term antitumor immune memory was induced in treated mice for preventing tumor recurrence.

By using gold nanoparticles as the NIR-II PTT agents, Wang’s group disclosed the NIR-II PTT to stimulate more compatible and deeper-tissue apoptosis of cancer cells and induce the immune response for the inhibition of tumor growth and distant metastasis [70]. The gold nanoparticles were self-assembled onto fluidic liposomes with different molar ratios to regulate the high-efficiency PTT transducers with precisely adjustable localized surface plasmon resonance. The corresponding photothermal conversion efficiency of gold-liposome nanoparticles was up to 20.94~23.66%, and they displayed excellent penetration at 1064 nm irradiation in a 4-cm-deep agarose gel. These nanoparticle-mediated NIR-II PTT (1.0 W/cm^2^, 10 min) could induce the formation and delivery of DAMP factors in breast-tumor cells in vivo through inducing ICD. Thus, the homogeneous release and distribution of DAMPs in the deeper regions of the tumors was formed to simultaneously trigger the innate and adaptive immune responses, thus enabling the efficient treatment of 4T1 tumors, with 5/8 of the mice remaining tumor-free in the cancer vaccination assay. Such a NIR-II PTT could be combined with anti-PD-1-mediated immune checkpoint blockade therapy to achieve long-term control of both primary and distant tumors.

The optical properties of gold nanoparticles can be modulated to obtain better antitumor efficacy. As an example, Huang’s group reported the synthesis of plasmonic modulating gold nanotheranostics modified with aptamers for targeted NIR-II PTT-augmented immunotherapy [71]. The features of the designed gold nanodumbbells (AuNDs) were adjusted by varying the contents and sequences of DNA chains, and their localized surface plasmon resonance was found to red-shift to the NIR-II window for efficient NIR-II PTT. The as-obtained AuNDs showed excellent photothermal conversion efficacy of 84.9% and good thermal stability. The nucleolin-targeted DNA aptamer AS1411 was conjugated with AuNDs to achieve tumor targeting and effective photoacoustic imaging-guided NIR-II PTT (1064 nm, 0.4 W/cm^2^, 5 min) for 4T1 tumors. The gold nanotheranostic-mediated targeted NIR-II PTT also significantly inhibited tumor growth and triggered a strong immune response in combination with the anti-PD-L1 antibody to kill the distant breast tumors and metastatic 4T1 cells throughout the whole body in vivo.

Duan’s group recently designed a plasmonic gold-nanoparticle-based mesoporous polydopamine (mPDA) core-shell structure to attain targeted transmission and controlled the release of DNase I for the local degeneration of neutrophil extracellular traps (NETs) in NIR-II-mediated PTT treatment for efficient colon tumor immunotherapy and strong metastasis inhibition [72]. The gold-mPDA nanostructures presented a homogenous core-shell structure and excellent photothermal ability under 1064 nm laser irradiation (0.33 W/cm^2^, 15 min). The nanostructures could be delivered into targeted sites of cancer cells and exerted NIR-II PTT to induce the ICD of cancer cells in vitro and in vivo. In addition, the NIR-II laser irradiation could trigger the on-demand release of DNase I to break the “NET-mediated physical barrier”, thus increasing the contact of immune cells with tumor cells to sensitize the immune checkpoint blockade therapy in living mice. The anti-PD-1 antibody was injected into tumors with the assistance of nanostructure-induced NETs digestion and NIR-II PTT therapy to treat orthotopic MC38 colorectal tumors and prevent MC38 colorectal cancer liver metastasis.

**Figure 4:**
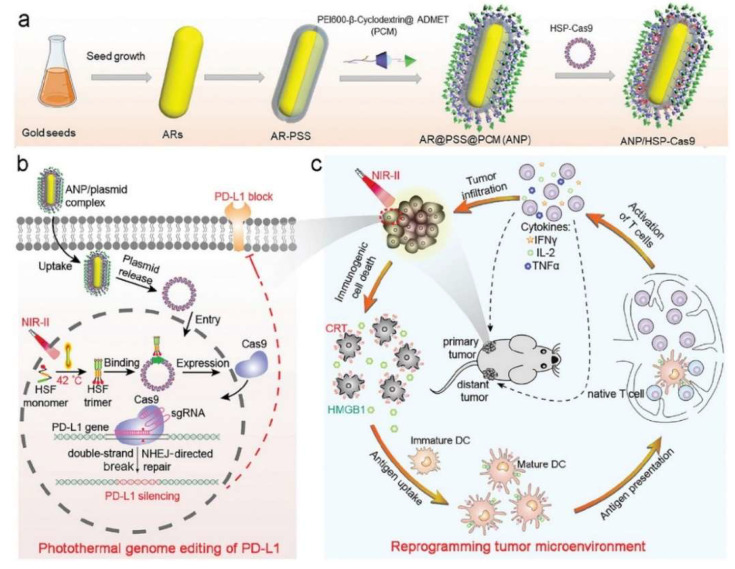
(**a**) A schematic illustration of the construction of the ANP/HSP-Cas9 plasmid nanocomplex. (**b**) A schematic illustration of the photothermal activation for PD-L1 genome editing in tumor cells under NIR-II laser irradiation. (**c**) A schematic illustration of the NIR-II photoactivable CRISPR/Cas9 strategy to reprogram the immunosuppressive tumor environment for enhanced cancer immunotherapy. Reproduced with permission from [73]. Copyright 2021, Wiley-VCH.

To improve the immune checkpoint blockade therapeutic efficacy via a genome-editing strategy, Ping’s group proposed a PTT-targeted PD-L1 genome-editing method through utilizing the supramolecular cationic AuNRs to reprogram the tumor immunosuppressive microenvironment for engendering efficient NIR-II PTT and immunotherapy [73]. This strategy was based on mild hyperthermia-induced genome editing using cationic gold nanoparticle-mediated transfection. The AuNRs were synthesized by CTAB-mediated preparation and coated with polystyrene sulfonate (PSS) and a supramolecular polymer of PCM (self-assembled between polyethyleneimine-modified β-cyclodextrin and biguanidyl admantane) to construct stable AuNRs complexes, followed by the loading of the CRISPR/Cas9 plasmid with a heat-inducible promoter (HSP) (Figure 4a). The cationic AuNRs could transform the NIR-II light (0.33 W/cm^2^, 30 min) into PTT-mediated hyperthermia to cause ICD and played a crucial role in delivering CRISPR/Cas9 targeting PD-L1 to activate the gene expression of Cas9 in tumor cells and single-guide RNA (sgRNA) targeting PD-L1 upon NIR-II laser irradiation, leading to the precise genome editing of PD-L1 on tumor cells (Figure 4b). The genome of PD-L1 was disrupted for significantly the augmented efficacy of immune checkpoint blockade therapy to increase the infiltration of T cells into tumor sites for reprogramming an immunosuppressive tumor condition into an immunoactive microenvironment (Figure 4c). Such a therapeutic modality, which involved combining NIR-II PTT with genome-editing-mediated immune checkpoint blockade therapy, greatly inhibited the growth of primary and distant metastatic tumors and exhibited long-term immune memory effects to suppress both rechallenged and recurrent tumors in B16F10 tumor-bearing mice.

NIR-II-induced PTT and photodynamic therapy (PDT), with the assistance of immune checkpoint blockade immunotherapy, is exploited to produce a high temperature and ROS to further enhance the efficiency of cancer therapy. Yang’s group reported a corn-shape Au/Ag nanorod (Au/Ag NR) system to mediate NIR-II PTT/PDT and potentiate the immune checkpoint blockade therapy efficacy via reprogramming the tumor immunosuppressive microenvironment [74]. Ag was decorated around the synthesized AuNR cores to grow the Ag shell for the preparation of corn-shape Au/Ag NRs, which showed efficient PTT and PDT effects with the generation of heat and ROS under 1064 nm laser irradiation (1.0 W/cm^2^, 10 min). Au/Ag NR-mediated PTT and PDT generated the ICD of 4T1 cancer cells in vitro and triggered antitumor immunity via reprogramming the immunosuppressive cold-tumor microenvironment in vivo. Anti-PD-1 and anti-CTLA-4 antibodies could be used to increase the synergistic therapeutic efficiency for distant tumor suppression after treatment with the Au/Ag NRs and NIR-II laser irradiation. In addition, Au/Ag NRs-induced PTT and PDT sensitized tumors to the anti-CTLA-4 antibody to trigger a long-term immune memory, leading to the inhibition of the lung metastasis of the 4T1 tumors and the protection of mice against tumor cell rechallenge 40 days post treatment.

### 4.2. Copper-Based Nanoparticles

Besides gold nanoparticles, copper-based nanoparticles have been widely utilized for synergistic NIR-II PTT and the immunotherapy of tumors. As an example, Yao’s group reported copper sulfide (CuS)-based NIR-II PTT to ablate solid tumors and provoke the release and delivery of interleukin-12 (IL-12) for eradicating primary and distant tumors [75]. The photothermal CuS nanoparticles were loaded inside the SiO_2_ pore channels with the surface polycation of poly((2-dimethylamino) ethyl methacrylate) (PDMAEMA) to carry the plasmid-encoding IL-12 gene. The CuS-mediated NIR-II PTT under 1064 nm laser irradiation (0.6 W/cm^2^, 5 min) produced hyperthermia to significantly kill local tumor cells, and the plasmid triggered the high expression of IL-12 to promote the maturation of DCs, and the division and infiltration of CD8^+^ T cells for the repression of metastatic B16F10 tumors.

Luo’s group reported CuS-based NIR-II PTT-amplified immunotherapy using photoactivatable composite nanostimulators [76]. Thermal-sensitive liposomes were constructed to load CuS as a NIR-II photothermal agent, cytosine-phospho-guanine oligodeoxynucleotides (CpG) as a TLR-9 agonist, and JQ1 as a PD-L1 inhibitor. Under 1064 nm laser irradiation (1.0 W/cm^2^, 5 min), CuS-mediated NIR-II PTT increased the temperature, not only leading to tumor ablation and the ICD effect but also causing the precise release of CpG and JQ1 into the tumor microenvironment through disrupting the thermal-responsive lipid shell. Via combining ICD action, TLR-9 stimulation, and the down-expression of PD-L1, the maturation of DCs was promoted and the infiltration of CTLs was increased, indicating the enhancement of the antitumor immune responses. Therefore, these nanostimulator-mediated NIR-II PTT-synergized immunotherapy efficiently suppressed the growths of primary and distant tumors in Panc02 and 4T1 tumor-bearing-mouse models and also prevented lung metastasis for 4T1 tumors.

Copper-based nanoparticles have also been developed for NIR-II laser-derived PTT and chemodynamic therapy (CDT) with the assistance of immunotherapy for repressing malignant tumors. Huang’s group recently reported a multifunctional Fenton Cu-based nanoparticle to mediate NIR-II PTT and CDT for enhancing the immunotherapy of the anti-PD-L1 antibody [77]. The plasmonic Cu_9_S_8_ self-doped nanoparticles with a hollow character enabled an excellent Fenton-like agent to generate an abundant amount of hydroxyl radicals (·OH) and exhibited strong localized surface plasmon resonance absorption in the NIR-II region for NIR-II PTT. In addition, 1064 nm laser irradiation (0.2 W/cm^2^, 5 min) could enhance the CDT effect based on plasmon-driven photoredox chemistry. Such a synergistic PTT and CDT resulted in the high mortality of the 4T1 tumor cells in vitro and the suppressive growth of the 4T1 tumors in vivo after the treatment of the Cu_9_S_8_ nanoparticles with 1064 nm laser irradiation. Furthermore, the intracellular ROS generated by the NIR-II laser-induced enhanced Fenton reaction induced ICD and stimulated the maturation of DCs both in vitro and in vivo. The antitumor immune response was greatly improved via the synergistic effect of ICD and the anti-PD-L1 antibody treatment. Such a combinational therapy allowed for the primary tumor elimination and the effective suppression of distant tumors and lung metastases for 4T1 tumors.

### 4.3. Iron-Based Nanoparticles

**Figure 5:**
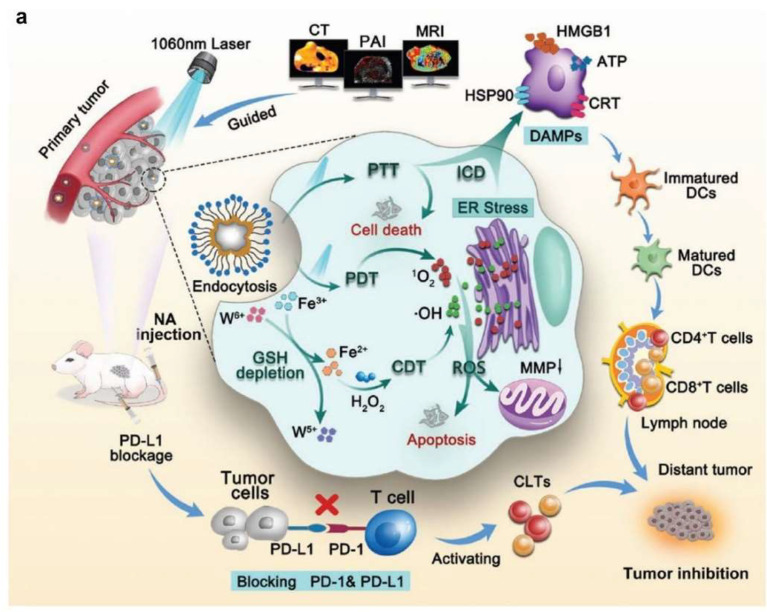

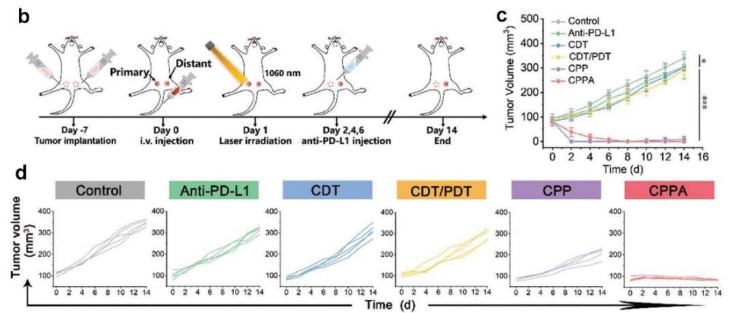
(**a**) A schematic illustration of FeWOx-PEG nanoadjuvant-mediated CDT/PDT/PTT under NIR-II laser irradiation and antitumor immunity effect amplification via combining ROS/PTT-triggered ICD with immune checkpoint blockade therapy under the imaging guidance. (**b**) A schematic diagram combining the FeWOx-PEG nanoadjuvant with the anti-PD-L1 strategy for inhibiting a distant tumor on female BALB/c mice. (**c**) The tumor volume changes of the primary tumor after various treatments. (**d**) The tumor volume changes of the distant tumor in six groups after various treatments. Reproduced with permission from [78]. Copyright 2022, Wiley-VCH. A *p*-value < 0.05 was considered statistically significant, * *p* < 0.05, *** *p* < 0.001.

Iron-based inorganic nanomedicines have been used to combine NIR-II PTT with immunotherapy for the effective treatment of cancer. For example, Wang’s group constructed an Fe-based nanoadjuvant to mediate CDT, PDT, and NIR-II PTT for combining immune checkpoint blockade therapy for enhanced antitumor immunity [78]. The Fe nanoadjuvant system was constructed by incorporating iron tungsten oxide (FeWOx)-based nanosheets with surface PEGylation (FeWOx-PEG) to serve as the Fenton-like agent for CDT and the photosensitizer for PTT and PDT under 1060 nm laser irradiation (1.0 W/cm^2^, 5 min). Through exerting CDT via the Fenton reaction to efficiently produce ·OH, NIR-II PTT, and PDT under NIR-II laser irradiation to produce heat and ROS, FeWOx-PEG ultimately resulted in a combinational action of CDT/PDT/PTT and induced ICD in 4T1 breast cancer cells (Figure 5a). Such a combinational therapy showed an obviously improved efficacy in eliminating primary tumors, and the further synergism with the immune checkpoint blockade therapy mediated by the anti-PD-L1 antibody could significantly inhibit the distant tumors in 4T1 tumor-bearing-mouse models (Figure 5b–d).

In another study, Song’s group reported the use of Fe-based nanomedicines for magnetic-targeted NIR-II PA and magnetic resonance (MR) imaging-guided NIR-II PTT-immunotherapy [79]. Iron oxide nanoparticles were anchored onto titanium disulfide (TiS_2_) nanosheets via DSPE-PEG self-assembly, and the formed nanoparticles showed excellent photothermal and PA/MR properties. The nanoparticles achieved high tumor accumulation (17.9% of injected dose) under an applied magnetic field to exhibit significantly increased PA and MR signals. Under 1064 nm laser irradiation (1.0 W/cm^2^, 5 min), NIR-II PTT was exerted to trigger ICD and thus promote antitumor immunity. The nanoparticles thus showed the magnetic-targeted NIR-II PTT treatment, which could be combined with anti-PD-1-mediated immunotherapy to inhibit the tumor recurrence and metastasis of 4T1 tumor-bearing-mouse models. After this combinational treatment, fewer metastatic nodules were observed in lungs, suggesting the long-term immune memory effects in a 24-day course.

**Figure 6:**
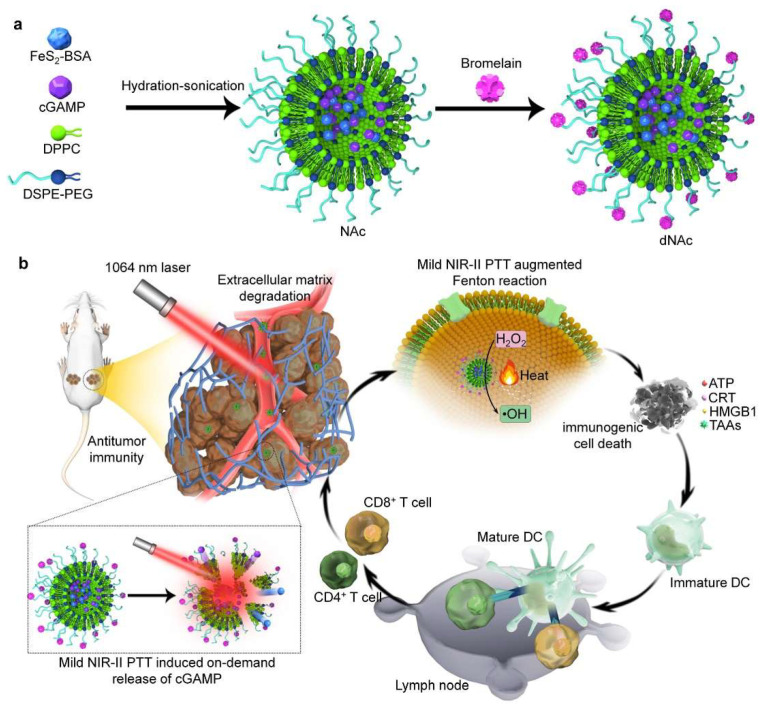
(**a**) A schematic illustration of the construction of ECM-degrading STING nanoagonists (dNAc) via hydration-sonication and the surface modification of bromelain. (**b**) A schematic illustration of the NIR-II photoactivation of dNAc for mild photothermal effect-augmented CDT-immunotherapy. Reproduced with permission from [80]. Copyright 2022, Springer Nature.

To promote immune cell infiltration into tumors and control the release of immunotherapeutic drugs for effective and safe cancer immunotherapy, our group reported an extracellular matrix (ECM)-degrading nanoagonist (dNAc) to enable mild NIR-II PTT-augmented CDT-immunotherapy [80]. The thermal-responsive liposomes with loadings of ferrous sulfide (FeS_2_) nanoparticles and 2′3′-cyclic guanosine monophosphate-adenosine monophosphate (cGAMP) were surface-modified with an ECM-degrading enzyme (bromelain) to obtain dNAc (Figure 6a). FeS_2_ nanoparticles acted as both the NIR-II photothermal converters and the Fenton catalysts, and cGAMP was used as an agonist of the stimulator of interferon genes (STING) for triggering antitumor immunity. Fenton reaction efficiency was enhanced for dNAc under 1064 nm laser irradiation (1.0 W/cm^2^, 10 min) due to the generation of mild heat, leading to the ICD induction of 4T1 cancer cells (Figure 6b). Furthermore, the generated heat triggered the controlled release of cGAMP from the thermal-responsive liposomes for the activation of the STING pathway. The surface-modified bromelain degraded the ECM in tumor tissues to further facilitate infiltrations of effector T cells into tumors after the combinational action of ICD and the mild photothermal activation of the STING pathway. The inhibition efficiency of primary tumors was as high as 97.9% for such a dNAc-mediated therapy, and the growth of distant tumors, and liver and lung metastasis, in 4T1 tumor-bearing-mouse models was effectively suppressed.

### 4.4. Other Inorganic Metal Nanoparticles

MXene, as a class of multifunctional two-dimension nanocrystals with optical absorbances in the NIR-II window, can produce hyperthermia and induce the ICD of cancer cells for combinational NIR-II PTT and immunotherapy. Based on the MXene family member niobium carbide (Nb_2_C), Liu’s group reported a combination of NIR-II PTT and immunotherapy to treat solid tumors [81]. Nb_2_C nanosheets with the surface modification of polydopamine layers were used to load R837, and the surface was further coated with the red blood cell membrane. R837 stimulated the maturation of DCs to trigger an antitumor immune response after the RBC membrane coating enabled the effective accumulation of nanoparticles into tumor sites. Under 1064 nm laser irradiation (1.5 W/cm^2^, 5 min), NIR-II PTT was exerted to kill primary tumors through thermal ablation with the release of antigens, which were presented to DCs with the assistance of released immunoadjuvant R837, leading to an enhanced immune response. After such a treatment, primary tumors were completely ablated in laser-irradiated sites, and the distant 4T1 tumors were also constrained for recurrence and metastasis.

In addition, Yuan’s group reported a temperature-feedback nanoplatform based on upconversion nanoparticles (UCNPs) and IR-1048 dye for NIR-II penta-modal imaging-guided synergistic PTT and the immunotherapy of lung cancer [82]. NaLuF_4_:Yb/Er@NaLuF_4_-based core-shell UCNPs and IR-1048 dye were loaded in lipid nanoparticles containing DSPE-PEG-AS1411 to construct multifunctional nanoplatforms via self-assembly. Within the nanoplatforms, the IR-1048 dye served as the theranostic agent for PA and photothermal imaging, optical coherence tomography angiography, and PTT, and the UCNPs acted as contrast agents for computed tomography (CT) and thermo-sensitive up-conversion luminescence (UCL) imaging. This enabled the real-time tracking of the metabolic activity of tumors and temperature-feedback PTT. These nanoplatforms mediated NIR-II PTT under 1064 nm laser irradiation (1.0 W/cm^2^, 6 min) to ablate the lung tumors, with minimal side effects because of the accurate monitoring of in situ temperature changes during PTT. Such a treatment could work in tandem with chimeric antigen receptorNK immunotherapy to eradicate residual tumor cells after PTT; thus, it shows great promising for the treatment of lung cancer.

## 5. Conclusions and Perspectives

Immunotherapy has provided a promising strategy for cancer treatment in both clinical practices and preclinical experiments, while its therapeutic efficacies are often unsatisfactory. The combination of nanomedicine-mediated NIR-II PTT and immunotherapy can be adopted to improve antitumor efficacy. This review summarizes the recent progress of engineering nanomedicines for the NIR-II PTT combinational immunotherapy of cancer while emphasizing the design principles of NIR-II-absorbing nanomaterials and their working mechanisms. The NIR-II-absorbing organic and inorganic non-metal and inorganic metal nanomedicines not only mediate the NIR-II PTT effect to directly ablate tumor cells but also trigger ICD, which can reverse the tumor immunosuppressive microenvironment and promote antitumor immunity. Therefore, the combinations of NIR-II PTT with immunotherapy often show enhanced efficacy at treating tumors. In addition, with the generated heat during NIR-II PTT, it is possible to realize the precision release of immunotherapeutic drugs in tumor tissues for safe and effective immunotherapy.

To promote the possible clinical translation of nanomedicine-enabled NIR-II PTT combinational immunotherapy, some crucial challenges need to be addressed. First, although the NIR-II light has shown an increased tissue penetration depth compared to the NIR-I light, it is still impossible for the NIR-II light to penetrate into deep tissues in the human body, which greatly obstructs the clinical applications of NIR-II PTT combinational immunotherapy [83]. By combining endoscopic light delivery technologies with nanomedicines, it is possible to achieve the NIR-II PTT combinational immunotherapy of deep-seated tumors [84]. Second, the controlled delivery of immunotherapeutic drugs in most examples should be able to be achieved as their uncontrolled distributions in living systems not only compromise antitumor efficacy but also cause severe immune-related adverse events. Utilizations of NIR-II light and/or tumor microenvironment-responsive drug delivery nanosystems potentially provide promising strategies for the precision delivery of drugs into tumor tissues, thus allowing for more safe and effective treatments of cancer [85,86,87,88,89]. Third, the long-term safety, biodegradability, and clearance of nanomaterials in living systems are very important to promote their clinical applications, which, however, remain a significant challenge. This concern should be addressed by constructing biodegradable or ultrasmall (size smaller than 5 nm) nanomedicines with NIR-II photothermal properties to ensure their rapid clearance after cancer treatment [90,91,92,93]. Fourth, the activation of immune responses is dynamic, and their monitoring is crucial to optimize the therapeutic window, which, however, has been poorly explored. The development of theranostic nanoplatforms through integrating imaging probes that show turn-on signals upon responding to the immune-specific biomarkers in NIR-II-absorbing nanomedicines is highly desired to achieve simultaneous cancer treatment and the monitoring of immune response activation [94,95,96]. Fifth, the tumor microenvironment is diverse and complicated, which will limit the efficacies of different immunotherapeutic strategies. The exploration of more immunotherapy targets can allow for the utilization of rational immunotherapeutic strategies for different types of tumors [97]. More effort should be made to address these concerns. In the future, nanomedicine-mediated NIR-II PTT combinational immunotherapy may play a vital role in the treatment of clinical tumors.

## Data Availability

The review is based on published data and sources of data, upon which conclusions have been drawn that can be found in the reference list.
